# Phase II Open-Label Study to Assess Efficacy and Safety of Lenalidomide in Combination with Cetuximab in *KRAS*-Mutant Metastatic Colorectal Cancer

**DOI:** 10.1371/journal.pone.0062264

**Published:** 2013-11-11

**Authors:** Salvatore Siena, Eric Van Cutsem, Mingyu Li, Ulf Jungnelius, Alfredo Romano, Robert Beck, Katia Bencardino, Maria Elena Elez, Hans Prenen, Mireia Sanchis, Andrea Sartore-Bianchi, Sabine Tejpar, Anita Gandhi, Tao Shi, Josep Tabernero

**Affiliations:** 1 Ospedale Niguarda Ca' Granda, Milan, Italy; 2 University Hospitals Leuven and KU Leuven, Leuven, Belgium; 3 Celgene Corporation, Summit, New Jersey, United States of America; 4 Vall d'Hebron University Hospital, Universitat Autònoma de Barcelona, Barcelona, Spain; Davidoff Center, Israel

## Abstract

This study aimed to assess the efficacy and safety of combination treatment with lenalidomide and cetuximab in *KRAS*-mutant metastatic colorectal cancer patients. This was a phase II multicenter, open-label trial comprising a safety lead-in phase (phase IIa) to determine the maximum tolerated dose, and a randomized proof of concept phase (phase IIb) to determine the response rate of lenalidomide plus cetuximab combination therapy. Phase IIa treatment comprised oral lenalidomide (starting dose 25 mg/day) and intravenous cetuximab (400 mg/m^2^ followed by weekly 250 mg/m^2^) in 28-day cycles. In phase IIb patients were randomized to either the phase IIa treatment schedule of lenalidomide plus cetuximab combination therapy or lenalidomide 25 mg/day monotherapy. Eight patients were enrolled into phase IIa. One patient developed a dose-limiting toxicity and the maximum tolerated dose of lenalidomide was determined at 25 mg/day. Forty-three patients were enrolled into phase IIb proof of concept. Best response was stable disease in 9 patients and study enrollment was terminated prematurely due to lack of efficacy in both treatment arms and failure to achieve the planned response objective. The majority of adverse events were grade 1 and 2. In both phases, the adverse events most commonly attributed to any study drugs were fatigue, rash and other skin disorders, diarrhea, nausea, and stomatitis. Thirty-nine deaths occurred; none was related to study drug. The combination of lenalidomide and cetuximab appeared to be well tolerated but did not have clinically meaningful activity in *KRAS*-mutant metastatic colorectal cancer patients.

**Trial Registration:**

Clinicaltrials.gov NCT01032291

## Introduction

It has been documented that colorectal cancer (CRC) is the third most common cancer in men and second in women worldwide, and around 10% of all malignancies are colorectal tumors [1]. Approximately 40–50% of these colorectal tumors have activating mutations in the *KRAS* (v-Ki-ras2 Kirsten rat sarcoma viral oncogene homolog) gene [2].

*KRAS* is involved in cell signaling pathways, including the signal transduction of the epidermal growth factor receptor (EGFR). Anti-EGFR monoclonal antibodies (mAbs), panitumumab (Vectibix®, Amgen Inc.) and cetuximab (Erbitux®, Merck KGaA), have demonstrated efficacy in wild-type *KRAS* metastatic CRC (mCRC) [3]–[6]. However, due to primary resistance these compounds have little or no efficacy in mCRC cells harboring *KRAS* mutations [7], [8]. For patients with *KRAS*-mutated mCRC that is resistant to or has relapsed after fluoropyrimidine-, oxaliplatin-, and irinotecan-containing therapies, treatment beyond best supportive care is very limited [9]. Several investigational agents, such as regorafenib [10] and perifosine [11], are currently under evaluation in mCRC. In a phase III study in mCRC patients, regorafenib showed significantly better overall survival (OS) and progression-free survival (PFS) than placebo; this benefit has been shown in the *KRAS*-mutated population as well [10]. In contrast, in a randomized phase II study perifosine in combination with capecitabine was suggested to be active in the refractory setting [11], although a recently presented phase III study has not been able to confirm these results [12].

Cetuximab is an anti-EGFR mAb that is indicated for treatment of *KRAS* wild-type mCRC [13]. In addition to immune system activation [14] and blockage of the EGFR signaling pathway [15], [16], many therapeutic mAbs also act through the mechanism of interaction of the Fcγ receptor (FcγR) with immune complexes triggering biological responses that include phagocytosis, release of inflammatory mediators, antibody dependent cellular cytotoxicity (ADCC), blockade of growth factor binding, enhancement of antigen presentation, and platelet activation [17]. Genetic variation in FcγRs is suggested to play an important role in disorders of the host defense system [18], immunohematologic disease [19], and systemic autoimmune disease [20], [21], as well as in the efficacy of mAbs [22], [23], at least for those that have an immunoglobulin (Ig)G1 structure.

Lenalidomide (Revlimid®, Celgene Corporation) is an immunomodulatory agent with antiangiogenic and antineoplastic properties that has demonstrated efficacy and an acceptable toxicity profile in multiple myeloma and myelodysplastic syndromes [24]–. Lenalidomide has also demonstrated antiangiogenic activity in a CRC model [27]. In mice, daily administration of lenalidomide reduced the rate of tumor growth significantly and during histological analysis of the tumors, vast areas of necrotic tissue were found [27].

In further preclinical studies, the combination of lenalidomide plus cetuximab caused lysis of CRC cells, including cells with *KRAS* mutations [28]. Lenalidomide enhanced natural killer (NK) cell-mediated lysis of CRC cells coated with cetuximab by ADCC [28]. Lysis of CRC cells was independent of *KRAS* mutational status since ADCC bypasses this defect in the proliferative pathways in the cell [28]. This effect was not observed with the combination of lenalidomide and panitumumab, this finding being justifiable by the fact that panitumumab is an IgG2 anti-EGFR mAb without ADCC-inducing capacity.

## Materials and Methods

### Study design

**Figure 1:**
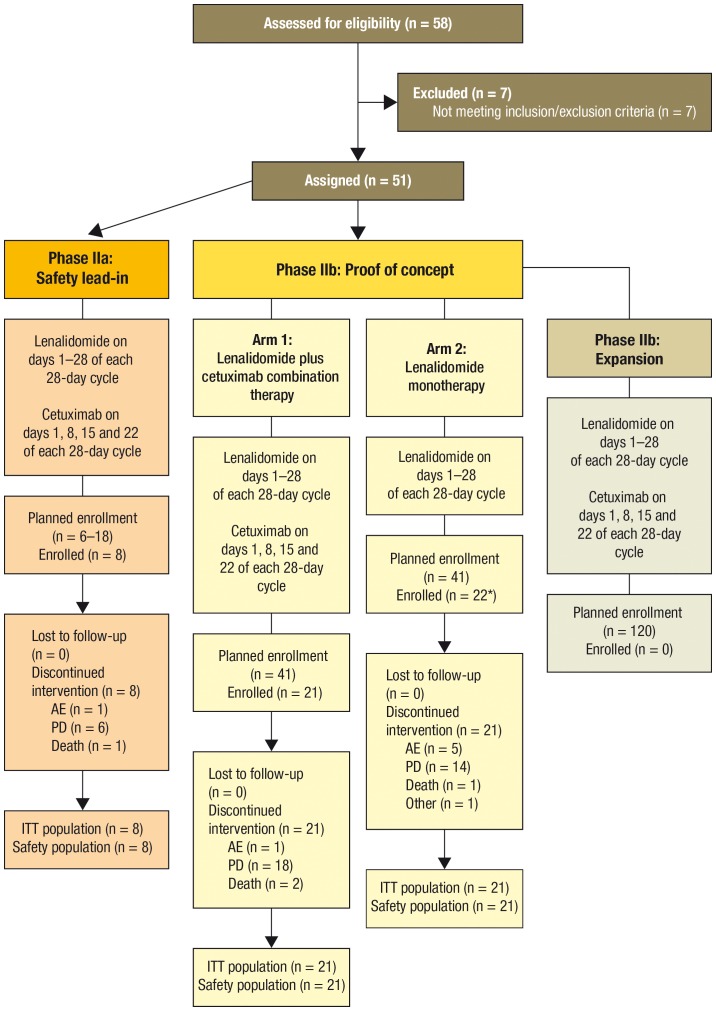
Study design and enrollment in patient groups. Study was terminated before the expansion part of phase IIb. *One patient was randomized to the lenalidomide monotherapy group but discontinued before taking any study drug and was therefore excluded from the analyses. AE, adverse event; ITT, intention to treat; PD, progressive disease.

This phase II, multicenter, open-label trial was conducted in accordance with the ethical principles of the Declaration of Helsinki and the Good Clinical Practice, according to the International Conference on Harmonization of Technical Requirements for Registration of Pharmaceuticals for Human Use. The study protocol, the proposed informed consent form, and other information to subjects, were approved by the Comitato Etico-Scientifico, Ospedale Niguarda Ca' Granda, Milan, Italy and properly constituted Institutional Review Boards/Independent Ethics Committees of all participating institutions. The protocol for this trial and supporting CONSORT checklist are available as supporting information; see Checklist S1 and Protocol S1. The trial design consisted of a safety lead-in phase (phase IIa) to determine the maximum tolerated dose (MTD) of lenalidomide when combined with cetuximab, and a randomized phase IIb to determine the response rate of the combination compared with lenalidomide as a single agent (Figure 1). Phase IIb consisted of a proof of concept (POC) part and an expansion part.

### Patients

Patients were eligible to participate in this study if they were diagnosed with metastatic colorectal adenocarcinoma with a confirmed *KRAS* mutation status. Patients must have progressed on oxaliplatin- and irinotecan-containing regimens, with at least one of these regimens containing bevacizumab. Eastern Cooperative Oncology Group performance status (ECOG PS) score of patients was ≤1. Written informed consent was obtained from all participants involved in the study.

### Objectives

The primary objectives of this trial were to determine the MTD and response rate of lenalidomide in combination with cetuximab. Secondary objectives were to establish the safety, tolerability, and clinical efficacy of the combination. Identifying biomarkers for validation of clinical efficacy and toxicity was an exploratory objective.

Adverse events (AEs) were graded using the National Cancer Institute Common Terminology Criteria for Adverse Events (NCI CTCAE) version 4.0 at each visit, with grade 5 representing deaths related to AEs. An AE was considered to be treatment-emergent (TEAE) if it occurred or worsened on or after the first treatment with the study drug, and within 28 days after the last dose was received. AEs were suspected to be related to the study drug if the temporal relationship of the AE to the administration of lenalidomide or cetuximab made a causal relationship possible, and other medications, therapeutic interventions, or underlying conditions did not provide a sufficient explanation for the observed event. All patients who received at least 1 dose of study drug were included in the safety analyses. Response rate and tumor progression were to be determined per Response Evaluation Criteria in Solid Tumors version 1.1.

### Phase IIa

A total of 6–18 patients were planned to be enrolled into phase IIa. Patients received oral lenalidomide at a starting dose of 25 mg daily. Cetuximab was administered as a 400 mg/m^2^ initial intravenous infusion, followed by 250 mg/m^2^ infusions on days 1, 8, 15, and 22 of each 28-day cycle. Dose escalation was not allowed and treatment was to be continued until tumor progression, unacceptable toxicity, death, or treatment discontinuation for any other reason.

The MTD of lenalidomide was defined as the highest dose level at which no more than 1 out of 6 patients experienced a dose-limiting toxicity (DLT). DLT was defined as missing at least 7 days of lenalidomide and/or 1 dose of cetuximab during the first cycle due to one or more of these drug-related AEs: any grade 3 or 4 non-hematological toxicity (excluding rash [treated and resolved according to guidelines] and alopecia); or grade 4 neutropenia, febrile neutropenia, or thrombocytopenia. If no more than 1 out of 6 subjects experienced a DLT in cycle 1, 25 mg lenalidomide would be the MTD. If 2 subjects or more experienced a DLT, then 6 additional subjects were to be enrolled at lower doses (20 mg) of lenalidomide. If 2 subjects or more experienced a DLT at this dose, the lenalidomide dose for the following 6 patients would be 15 mg. If no more than 1 patient experienced a DLT, that dose was determined to be the MTD. Patients experiencing a DLT were allowed to continue treatment at a lower dose of lenalidomide.

### Phase IIb

In the POC part of phase IIb, 82 patients were planned to be randomized at a 1∶1 ratio to either continue the phase IIa treatment schedule with lenalidomide at the MTD or to receive oral lenalidomide monotherapy dosed at 25 mg/day. Proceeding to the expansion part of phase IIb would be possible if the response rate from either arm in the POC part was significantly more than 10%. In the expansion part, patients were to be treated with the phase IIa treatment schedule with lenalidomide at the MTD.

### Follow-up

Patients were to be followed-up with one visit 28 days after the last dose of study drug was administered and subsequent telephone contacts for survival every 90 days until death or 5 years post-discontinuation.

### Biomarker analysis

A biomarker and pharmacodynamic marker analysis including FcγR genotyping, EGFR copy number, and immunomodulation was performed in this study. FcγR polymorphisms were determined in paraffin tumor samples by Genoptix Medical Laboratory (Carlsbad, CA) by DNA sequencing with allele-specific polymerase chain reaction serving as a back-up assay when required. *EGFR* gene copy number was analyzed in the Genoptix Medical Laboratory on individual patients' tumor specimens using a standard, validated fluorescent *in situ* hybridization (FISH) protocol. It was scored by a pathologist as positive or negative for *EGFR* amplification based on the following predetermined criteria: positive if there were more than 4 *EGFR* copies in at least 40% of cells or if the *EGFR*/chromosome 7 enumeration probe (CEP7) ratio was >2 in over 10% of cells; negative if the ratio *EGFR*/CEP7 was <2 in over 90% of cells or there were less than 4 *EGFR* copies in more than 60% of cells.

### Sample size

During phase IIa, up to approximately 18 subjects were to be enrolled. During the phase IIb POC part, up to approximately 82 subjects were to be randomized in a 1∶1 ratio between the lenalidomide plus cetuximab combination therapy arm and the lenalidomide monotherapy arm. A Simon two stage minimax design was used to monitor subject enrollment for each randomization arm separately. In the first stage, 23 subjects were to be enrolled. If 2 or less of the 23 subjects (<10%) had a response in either arm, the enrollment for that arm was to be stopped. If more than 2 of the 23 subjects had a response in either arm, the enrollment in that arm was to be continued until 41 subjects were enrolled. If one arm would be stopped, all new subjects were to be enrolled in the remaining arm. At the final analysis, the regimen would be concluded with a more than 10% true response rate if 9 or more of 41 subjects (>21%) have a response. This design had 90% power to conclude the true response rate higher than 10% at one-sided 2.5% level when the true response rate is 30%.

When any arm from the phase IIb POC was considered positive, the study would proceed with that regimen to the phase IIb Expansion phase. In the Expansion phase, approximately 120 subjects were to be treated with the regimen. This sample size would allow for a two sided 95% confidence interval of (22%, 39%) when 30% response rate is observed.

## Results

### Patient disposition

A total of 8 patients were enrolled into phase IIa of the study (Figure 1). In the phase IIb POC, 43 patients were enrolled; 1 patient did not take any study drug and was therefore excluded from the analyses.

### Baseline characteristics

During the phase IIa, the median age was 60.5 years, all patients were of Caucasian origin, there were equal numbers males and females, and in 75% of patients the ECOG PS score was 0 at baseline (Table 1).

**Table 1:** Baseline characteristics phase IIa and IIb.

Characteristic		Phase IIa	Phase IIb		
		Lenalidomide+cetuximab (n = 8)	Lenalidomide (n = 21)	Lenalidomide+cetuximab (n = 21)	Overall (N = 42)
Age, years	Median (range)	60.5 (45.0–70.0)	54.0 (38.0–75.0)	57.0 (31.0–70.0)	56.0 (31.0–75.0)
	≤65 years	7 (87.5%)	16 (76.2%)	18 (85.7%)	34 (81.0%)
	>65 years	1 (12.5%)	5 (23.8%)	3 (14.3%)	8 (19.0%)
Sex	Male	4 (50.0%)	12 (57.1%)	12 (57.1%)	24 (57.1%)
	Female	4 (50.0%)	9 (42.9%)	9 (42.9%)	18 (42.9%)
Race	Caucasian	8 (100%)	21 (100%)	20 (95.2%)	41 (97.6%)
	Non-caucasian	0	0	1 (4.8%)	1 (2.4%)
ECOG PS score	0	6 (75.0%)	10 (47.6%)	14 (66.7%)	24 (57.1%)
	1	2 (25.0%)	10 (47.6%)	7 (33.3%)	17 (40.5%)
	2	0	1 (4.8%)	0	1 (2.4%)
KRAS mutations^a^	12ASP	3 (37.5%)	5 (23.8%)	8 (38.1%)	13 (31.0%)
	12VAL	2 (25.0%)	5 (23.8%)	3 (14.3%)	8 (19.0%)
	12CYS	1 (12.5%)	2 (9.5%)	4 (19.0%)	6 (14.3%)
	13ASP	0	2 (9.5%)	1 (4.8%)	3 (7.1%)
	12ALA	0	2 (9.5%)	0	2 (4.8%)
	12ARG	0	0	2 (9.5%)	2 (4.8%)
	12SER	0	1 (4.8%)	0	1 (2.4%)
	N.a.^b^	2 (25.0%)	2 (9.5%)	2 (9.5%)	4 (9.5%)
	Negative^b^	0	2 (9.5%)	1 (4.8%)	3 (7.1%)

a *KRAS* mutations as determined by Genoptix Medical Laboratory.

b *KRAS* mutation found in local laboratory.

Abbreviations: ECOG PS: Eastern Cooperative Oncology Group performance status; n.a.: not applicable.

The median age of the patients enrolled into phase IIb was 56 years, 97.6% were Caucasian and 57.1% were male. Baseline ECOG PS score was 0 (57.1%) or 1 (40.5%), and 1 patient had a score of 2. Types of *KRAS* mutations included 12ASP, 12VAL, 12CYS, 13ASP, 12ALA, 12ARG, and 12SER. The lenalidomide plus cetuximab combination therapy arm and lenalidomide monotherapy arm were well matched for demographics and baseline characteristics.

### Maximum tolerated dose

In phase IIa, 1 patient developed a DLT: a grade 3 hypersensitivity reaction to cetuximab which led to permanent withdrawal of cetuximab therapy during Cycle 1. As this was the only DLT in phase IIa, the MTD was determined at 25 mg lenalidomide per day. This was used as the dose for phase IIb.

### Safety

As exposure to cetuximab and lenalidomide was comparable between the two phases of the study, safety results of both phases are combined in this section.

#### Treatment-emergent adverse events

All 29 patients on the combination regimen and 20 of the 21 patients on lenalidomide monotherapy experienced at least one TEAE. Overall, 54% and 93% of patients experienced at least 1 TEAE that was suspected by the investigator to be related to lenalidomide or cetuximab, respectively (Table 2). The most common AEs related to lenalidomide were fatigue (26%), rash (26%), pruritus (12%), and diarrhea and nausea (10% each). These AEs occurred more frequently in patients treated with the lenalidomide plus cetuximab combination regimen than with lenalidomide monotherapy. AEs most commonly attributed to cetuximab were rash (59%), fatigue (31%), dry skin (28%), and erythema, skin fissures, pruritus and stomatitis (14% each).

**Table 2:** TEAEs suspected by the investigator to be related to study drug, occurring in ≥2 patients, sorted by overall incidence.

	Phase IIa		Phase IIb		
	Lenalidomide+cetuximab (n = 8)		Lenalidomide (n = 21)	Lenalidomide+cetuximab (n = 21)	
	Related to lenalidomide	Related to cetuximab	Related to lenalidomide	Related to lenalidomide	Related to cetuximab
Patients with ≥1 TEAE related to study drug	5 (62.5%)	8 (100%)	9 (42.9%)	13 (61.9%)	19 (90.5%)
TEAEs grade ≥3^a^	4 (50.0%)	6 (75.0%)	6 (28.6%)	5 (23.8%)	12 (57.1%)
Rash	2 (25.0%)	5 (62.5%)	4 (19.0%)	7 (33.3%)	12 (57.1%)
Fatigue	3 (37.5%)	3 (37.5%)	3 (14.3%)	7 (33.3%)	6 (28.6%)
Dry skin	1 (12.5%)	3 (37.5%)	0	2 (9.5%)	5 (23.8%)
Pruritus	2 (25.0%)	2 (25.0%)	2 (9.5%)	2 (9.5%)	2 (9.5%)
Diarrhea	2 (25.0%)	1 (12.5%)	0	3 (14.3%)	2 (9.5%)
Stomatitis	1 (12.5%)	2 (25.0%)	0	1 (4.8%)	2 (9.5%)
Erythema	1 (12.5%)	2 (25.0%)	0	0	2 (9.5%)
Nausea	1 (12.5%)	0	1 (4.8%)	3 (14.3%)	0
Skin fissures	0	1 (12.5%)	0	1 (4.8%)	3 (14.3%)
Dyspnea	1 (12.5%)	1 (12.5%)	0	2 (9.5%)	0
Pyrexia	0	1 (12.5%)	1 (4.8%)	1 (4.8%)	1 (4.8%)
Anorexia	2 (25.0%)	1 (12.5%)	0	0	0
Hypokalemia	1 (12.5%)	1 (12.5%)	0	1 (4.8%)	0
Paronychia	0	3 (37.5%)	0	0	0
Hypomagnesemia	0	1 (12.5%)	0	1 (4.8%)	1 (4.8%)
Dysgeusia	0	0	1 (4.8%)	1 (4.8%)	1 (4.8%)
Mucosal inflammation	0	0	0	2 (9.5%)	1 (4.8%)
Vomiting	1 (12.5%)	0	0	1 (4.8%)	1 (4.8%)
Neutropenia	0	0	1 (4.8%)	1 (4.8%)	0
General physical health deterioration	1 (12.5%)	1 (12.5%)	0	0	0
Arthralgia	1 (12.5%)	1 (12.5%)	0	0	0
Photosensitivity reaction	1 (12.5%)	1 (12.5%)	0	0	0
Hypersensitivity	0	1 (12.5%)	0	0	1 (4.8%)
Skin hyperpigmentation	0	1 (12.5%)	0	0	1 (4.8%)
Hypertrichosis	0	1 (12.5%)	0	0	1 (4.8%)
Tachycardia	0	1 (12.5%)	0	1 (4.8%)	0
Vulvovaginal mycotic infection	0	0	0	1 (4.8%)	1 (4.8%)
Enteritis	0	0	0	1 (4.8%)	1 (4.8%)
Muscle spasms	0	0	0	1 (4.8%)	1 (4.8%)
Headache	0	0	0	2 (9.5%)	0

a All grade ≥3 TEAEs related to study drug were grade 3. Grade 3 events related to lenalidomide: fatigue (7; 2 events in 2 patients each), neutropenia (2), anorexia, hypokalemia, general physical health deterioration, diarrhea, increased gamma-glutamyltransferase, and decreased blood potassium. Grade 3 events related to cetuximab: rash (4; 3 events in 1 patient), fatigue (4; 2 events in 1 patient), hypersensitivity (3; 2 events in 1 patient), diarrhea (2), urticaria, general physical health deterioration, tachycardia, dyspnea, and hypertension.

All TEAEs that were deemed related to one of the study drugs were grade 3 or less. In 30% of patients AEs led to withdrawal of lenalidomide and in 34% led to dose reductions or interruptions of lenalidomide; withdrawal of cetuximab occurred in 31% of patients and dose reductions or interruptions of cetuximab were recorded in 28% of patients.

Fifteen patients had abnormal laboratory values that were reported as AEs, with an equal frequency in the lenalidomide monotherapy and lenalidomide plus cetuximab combination treatment groups. These AEs were mostly related to liver function and included increases in bilirubin, alanine aminotransferase, aspartate aminotransferase, alkaline phosphatase (ALP), and lactate dehydrogenase.

#### Severity of events

Overall, the majority of the TEAEs were NCI CTCAE grades 1 and 2. In the lenalidomide plus cetuximab combination therapy groups, 55% of patients experienced an AE of grade 3 or more, and in the lenalidomide monotherapy group this was 62%. Three grade 4 AEs occurred in the lenalidomide monotherapy arm (constipation, hyperbilirubinemia, and increased blood ALP); none occurred in the lenalidomide plus cetuximab combination therapy arms.

A total of 46% of patients experienced a serious AE (SAE); 43% of the lenalidomide monotherapy group and 48% of the lenalidomide plus cetuximab combination treatment groups. SAEs reported in more than 1 patient were general physical health deterioration (5 patients; 3 patients in the lenalidomide plus cetuximab combination treatment arms and 2 patients in the lenalidomide monotherapy arm), diarrhea, hyperbilirubinemia, and dyspnea (2 patients; both in the lenalidomide plus cetuximab combination treatment arms), abdominal pain (2 patients; both in the lenalidomide monotherapy arm), asthenia, infection, and pyrexia (1 patient in the lenalidomide monotherapy treatment arm and 1 patient in the lenalidomide plus cetuximab combination treatment arm).

#### Mortality

Thirty-nine patients (78%) died during the study. Three patients had causes of death listed other than disease progression (pulmonary embolism, respiratory failure, and septic shock), though the pulmonary embolism and respiratory failure were later found to be associated with disease progression.

Nine deaths were reported as grade 5 AEs. Five grade 5 AEs occurred in the lenalidomide monotherapy arm (metastasis to liver, metastasis to lung, large intestine perforation, and 2 events of general physical health deterioration) and 4 in the combination therapy arms (pulmonary embolism, general physical health deterioration, septic shock, and respiratory failure).

### Treatment duration and exposure

In phase IIa, the median duration of lenalidomide treatment was 8.1 weeks with a median dose intensity of 25 mg/day and a median relative dose intensity of 100% (Table 3). The median duration of cetuximab treatment was 7.1 weeks, with a median dose intensity of 287.9 mg/m^2^/week and a median relative dose intensity of 115.1%.

**Table 3:** Median treatment duration, cumulative dose, dose intensity, and relative dose intensity.

	Phase IIa		Phase IIb		
	Lenalidomide+cetuximab (n = 8)		Lenalidomide (n = 21)	Lenalidomide+cetuximab (n = 21)	
Median (range)	Lenalidomide exposure	Cetuximab exposure	Lenalidomide exposure	Lenalidomide exposure	Cetuximab exposure
Treatment duration (weeks)^a^	8.1 (4–16.4)	7.1 (2.1–16)	8 (0.7–33)	8 (2.3–24.1)	7.1 (1.3–23.1)
Cumulative dose (mg or mg/m^2^)^b^	1,412.5 (675–2,875)	1,897.8 (900–4,400)	1,375 (125–4,875)	1,400 (400–3,875)	2,148.4 (650–5,400)
Dose intensity (mg/week or mg/m^2^/week)^c^	175 (169–175)	288 (268–420)	175 (108–175)	175 (131–175)	301 (203–506)
Relative dose intensity (%)^d^	100 (96–100)	115.1 (107–168)	100 (61–100)	100 (75–100)	120.4 (81–202)

a Treatment duration = [(the study treatment end date)−(the first study drug start date)+1]/7.

b Cumulative dose = total doses taken during treatment phase.

c Dose intensity = cumulative dose/treatment duration.

d Relative dose intensity = dose intensity/planned dose intensity×100.

In phase IIb, the median duration of lenalidomide treatment was 8 weeks with a median dose intensity of 25 mg/day for both treatment arms. The median relative dose intensity was 100% in both arms. The median duration of cetuximab treatment was 7.1 weeks, with a median dose intensity of 301 mg/m^2^/week and a median relative dose intensity of 120.4%.

### Efficacy

Based on Biomedical System Corporation imaging data, the best response was stable disease in 9 patients, 1 patient from phase IIa and 8 patients from phase IIb, 5 of whom were treated with lenalidomide plus cetuximab combination treatment and 3 of whom were treated with lenalidomide monotherapy. Enrollment for the trial was, therefore, stopped, and the study was terminated prior to the expansion part of phase IIb due to lack of efficacy in any of the treatment arms and failure to achieve the planned response objective. Therefore, further efficacy and survival information were not collected and the secondary efficacy endpoints (PFS, response duration, disease control rate, and OS) were not analyzed.

**Figure 2:**
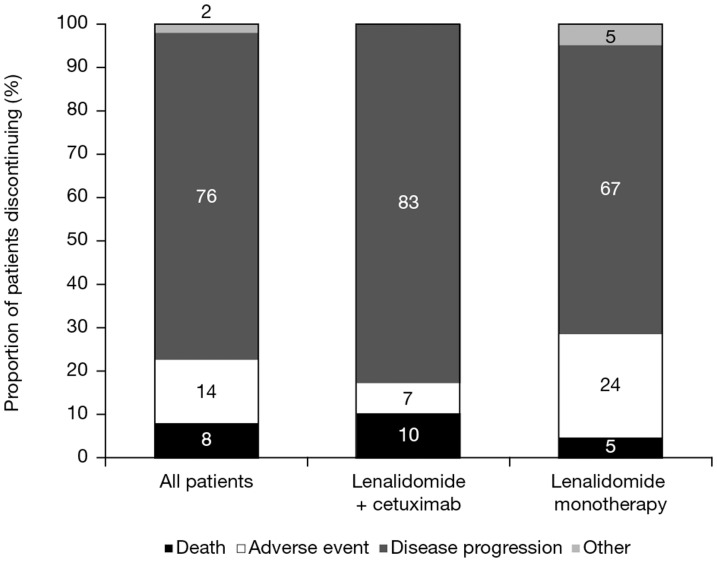
Reasons for discontinuation. One patient in the lenalidomide monotherapy group of phase IIb discontinued on investigator's decision due to lack of efficacy, mentioned as “other” in the figure.

All patients discontinued the study (Figure 2). In phase IIa, the reasons for discontinuation were disease progression in 6 patients, AEs in 1 patient, and death in 1 patient. In phase IIb, discontinuation was related to disease progression (76.2%; 66.7% in the lenalidomide monotherapy arm and 85.7% in the lenalidomide plus cetuximab combination therapy arm), AEs (14.3%), death (7.1%), and other reasons (2.4%).

### Biomarker analysis

A total of 27 subjects treated with lenalidomide plus cetuximab (from both phases IIa and IIb) have been analyzed for the correlative analysis of FcγR genotyping and OS–defined as the time from the date of randomization to the date of death (any cause). Patients that did not die were censored at the last known time the patient was alive. Kaplan-Meier curves were plotted and a log-rank p value was used to test the difference in OS between genotype groups (Figure S1 and Figure S2). No significant difference in OS was observed between the different genotype groups defined by either FcγRIIA or IIIA among these 27 subjects (log-rank p value >0.05). In contrast, the correlative analysis of EGFR gene copy number status (FISH-positive or FISH-negative) and OS in the 23 subjects who received lenalidomide plus cetuximab combination therapy showed that OS was significantly longer in EGFR FISH-positive than in EGFR FISH-negative subjects (log-rank p value = 0.0294) (Figure S3). Similar results were observed in the 20 subjects who received lenalidomide monotherapy (log-rank p value = 0.0582) (Figure S4). Significant immunomodulating effects were observed and a separate report on the immunomodulatory effects is forthcoming.

## Discussion

Currently, the National Comprehensive Cancer Network clinical practice guidelines [9] recommend *KRAS*-mutant CRC patients with metastatic or advanced disease to be treated with chemotherapy regimens including oxaliplatin, leucovorin, 5-fluorouracil, bevacizumab, capecitabine, and irinotecan. These compounds are used in both first- and second-line treatment regimens [9]. However, if patients are unsuitable for intensive therapy, treatment options are either 5-fluorouracil combined with leucovorin, or capecitabine with or without bevacizumab. If patients relapse or their functional status does not improve with treatment, the remaining treatment option is best supportive care [9]. This is the first study to assess the safety and efficacy of a combination regimen with cetuximab and lenalidomide in previously treated *KRAS*-mutant mCRC patients.

Lenalidomide has shown to significantly reduce tumor growth and cause necrosis in tumors in a CRC mouse model [27]. Additionally, when lenalidomide was combined with cetuximab in a CRC cell line, it enhanced NK cell-mediated lysis of CRC cells by ADCC, independent of *KRAS* mutational status [28]. Following these preclinical results, we hypothesized that the combination of lenalidomide and cetuximab could be active in patients with *KRAS*-mutated mCRC.

In the phase IIa part of this study, the median relative dose intensity was 100% for lenalidomide and 115.1% for cetuximab, even though no dose escalation was allowed in this phase of the trial. This is likely due to the relatively short treatment duration after the initial cetuximab dose of 400 mg/m^2^ and the fact that the relative dose intensity is defined as the dose intensity divided by the planned dose intensity, where the dose intensity is cumulative dose divided by treatment duration, and the planned dose intensity was 250 mg/m^2^/week.

With this dose intensity, only 1 DLT was reported during the dose finding phase and lenalidomide 25 mg/day was determined to be the MTD. The safety profile for lenalidomide and cetuximab in this study is consistent with that observed for lenalidomide in other non-hematological malignancies [29]–[31] and with that reported for cetuximab in this patient population [13]. In phase IIb of this study, stable disease was the best response seen in both the lenalidomide monotherapy and the lenalidomide plus cetuximab combination therapy arms. Therefore, enrollment was stopped prematurely. Safety and tolerability were evaluated throughout the study, but additional efficacy information for enrolled patients was not collected.

There are three distinct classes of FcγR that bind the Fc portion of IgG: FcγRI (CD64), FcγRII (CDw32), and FcγRIII (CD16). The FcγRII receptor is an immunoglobulin expressed on the surface of macrophages and neutrophils. A single nucleotide polymorphism (SNP) found in FcγRIIA results in two allotypes with either arginine (R) or histidine (H) at codon 131 [17]. The FcγRIIA 131 H/H has been found to bind IgG1 with a higher affinity than R/R homozygotes and H/R or R/H heterozygotes. The FcγRIIIA receptor is an immunoglobulin expressed on the surface of NK cells, monocytes, some T cells, and macrophages. A SNP found in FcγRIIIA results in two allotypes with either valine (V) or phenylalanine (F) at codon 158. IgG binds in the region proximal to amino acid 158, and the FcγRIIIA 158 V/V has been found to bind IgG1 with a higher affinity than F/F homozygotes and V/F or F/V heterozygotes. These polymorphisms at FcγRIIA codon 131 and FcγRIIIA codon 158 may play a role in immune activation and explain the variability of cetuximab-mediated clinical responses [32], [33]. In this study, the correlative analysis of OS and FcγRIIA or IIIA genotype showed that OS did not correlate with any particular genotype. This lack of correlation may support the finding that any prolongation of OS observed in the study population was not associated with lenalidomide enhancing ADCC because the binding affinity of cetuximab played no role in predicting OS.

The biomarker analysis showed that in patients with EGFR FISH-positive tumors, *i.e.*, CRC with increased *EGFR* gene copy number, OS was significantly longer than in EGFR FISH-negative subjects. Therefore, *EGFR* copy number might be of value as a prognostic marker. For subjects treated with lenalidomide plus cetuximab combination therapy, this finding is consistent with published reports of improved survival in patients with mCRC and high *EGFR* copy numbers who receive treatment with cetuximab [34]–[36]. However, the observation of an association between high *EGFR* copy number and OS in the lenalidomide monotherapy arm is inconclusive because of the small sample size. It should also be considered that assessment of *EGFR* gene copy number by FISH may be hampered by difficulties in clinical inter-laboratory reproducibility [37].

In conclusion, the combination regimen appeared to be well-tolerated and the toxicity profile of lenalidomide plus cetuximab combination therapy was similar to that of lenalidomide monotherapy. However, the short duration of exposure and small patient numbers limit drawing a definitive conclusion regarding safety. Despite preclinical evidence, present clinical data suggest the modulating effect of lenalidomide is unable to overcome primary resistance of *KRAS*-mutant mCRC to EGFR targeted inhibition by cetuximab. The combination of lenalidomide and cetuximab does not appear to demonstrate clinically meaningful activity in the treatment of *KRAS*-mutant mCRC patients.

## Supporting Information

Figure S1 FcγRIIA genotype and overall survival (OS) in the lenalidomide plus cetuximab combination therapy arm. There are no significant differences in OS among the three genotype groups for FcγRIIA. The median OS is 194, 129, and 152 days for the “H/H”, “H/R”, and “R/R” groups, respectively.
(TIF)

Figure S2 FcγRIIIA genotype and overall survival (OS) in the lenalidomide plus cetuximab combination therapy arm. There are no significant differences in OS among the three genotype groups for FcγRIIIA. The median OS is 176, 152, and 111 days for the “F/F”, “F/V”, and “V/V” groups, respectively.
(TIF)

Figure S3 Epidermal growth factor receptor (*EGFR*) copy number and overall survival (OS) in the lenalidomide plus cetuximab combination therapy arm. In the lenalidomide plus cetuximab combination therapy arm, OS was significantly shorter for *EGFR* FISH-negative than for *EGFR* FISH-positive subjects (median OS: 150 and >336 days, respectively). One subject whose *EGFR* status was tested twice by Genoptix had a negative and a positive result, and was considered *EGFR* FISH-positive for this analysis.
(TIF)

Figure S4 Epidermal growth factor receptor (*EGFR*) copy number and overall survival (OS) in the lenalidomide monotherapy arm. In the lenalidomide monotherapy arm, OS was shorter for *EGFR* FISH-negative than for *EGFR* FISH-positive subjects (median OS: 86 and >277 days, respectively).
(TIF)

Checklist S1 CONSORT Checklist.
(DOC)

Protocol S1 Trial Protocol.
(PDF)
