# Supplementary figures and images for: Phase II Open-Label Study to Assess Efficacy and Safety of Lenalidomide in Combination with Cetuximab in KRAS-Mutant Metastatic Colorectal Cancer
Source: PLoS One. 2013 Nov 11;8(11):e62264. doi: 10.1371/journal.pone.0062264 (PMC3823943; doi:10.1371/journal.pone.0062264)

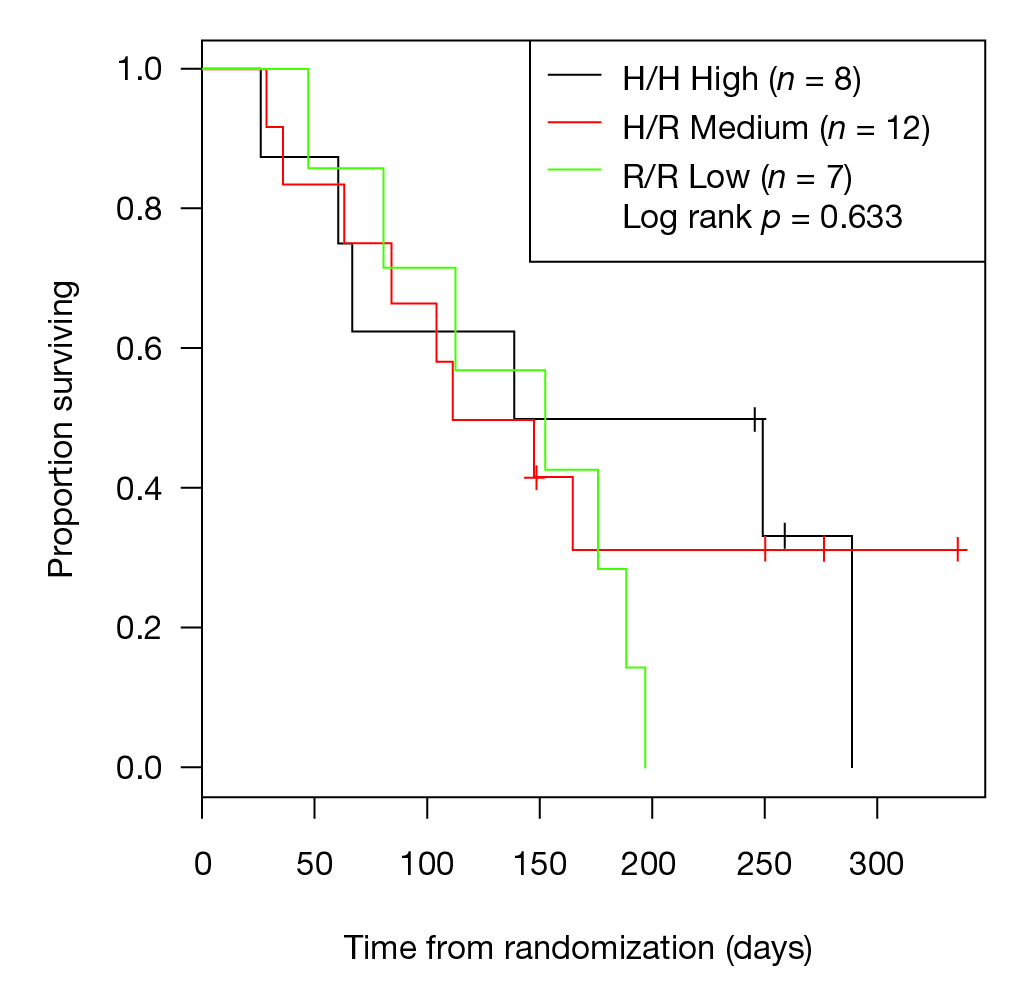

Supplement: Figure S1 — FcγRIIA genotype and overall survival (OS) in the lenalidomide plus cetuximab combination therapy arm. There are no significant differences in OS among the three genotype groups for FcγRIIA. The median OS is 194, 129, and 152 days for the “H/H”, “H/R”, and “R/R” groups, respectively. (TIF) [file pone.0062264.s001.tif]

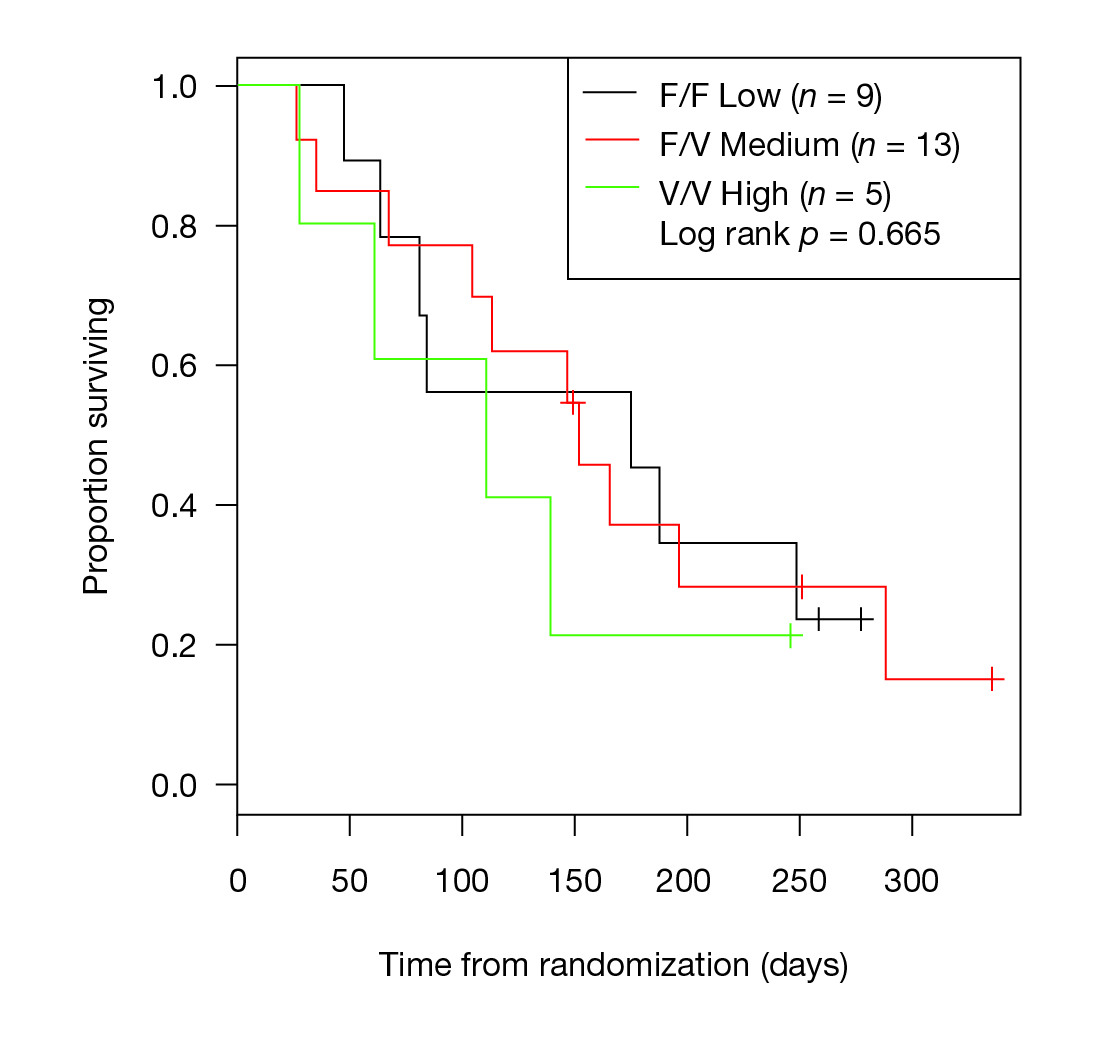

Supplement: Figure S2 — FcγRIIIA genotype and overall survival (OS) in the lenalidomide plus cetuximab combination therapy arm. There are no significant differences in OS among the three genotype groups for FcγRIIIA. The median OS is 176, 152, and 111 days for the “F/F”, “F/V”, and “V/V” groups, respectively. (TIF) [file pone.0062264.s002.tif]

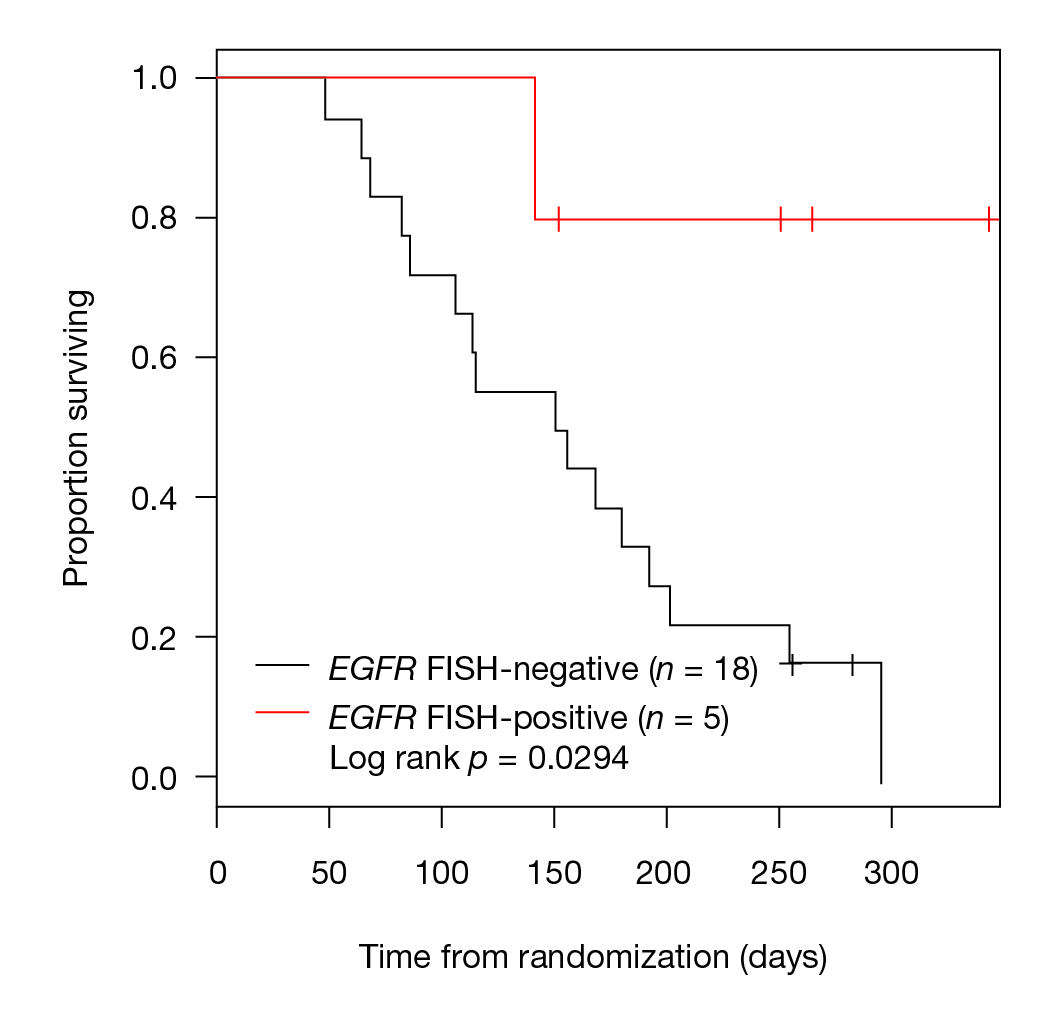

Supplement: Figure S3 — Epidermal growth factor receptor (EGFR) copy number and overall survival (OS) in the lenalidomide plus cetuximab combination therapy arm. In the lenalidomide plus cetuximab combination therapy arm, OS was significantly shorter for EGFR FISH-negative than for EGFR FISH-positive subjects (median OS: 150 and >336 days, respectively). One subject whose EGFR status was tested twice by Genoptix had a negative and a positive result, and was considered EGFR FISH-positive for this analysis. (TIF) [file pone.0062264.s003.tif]

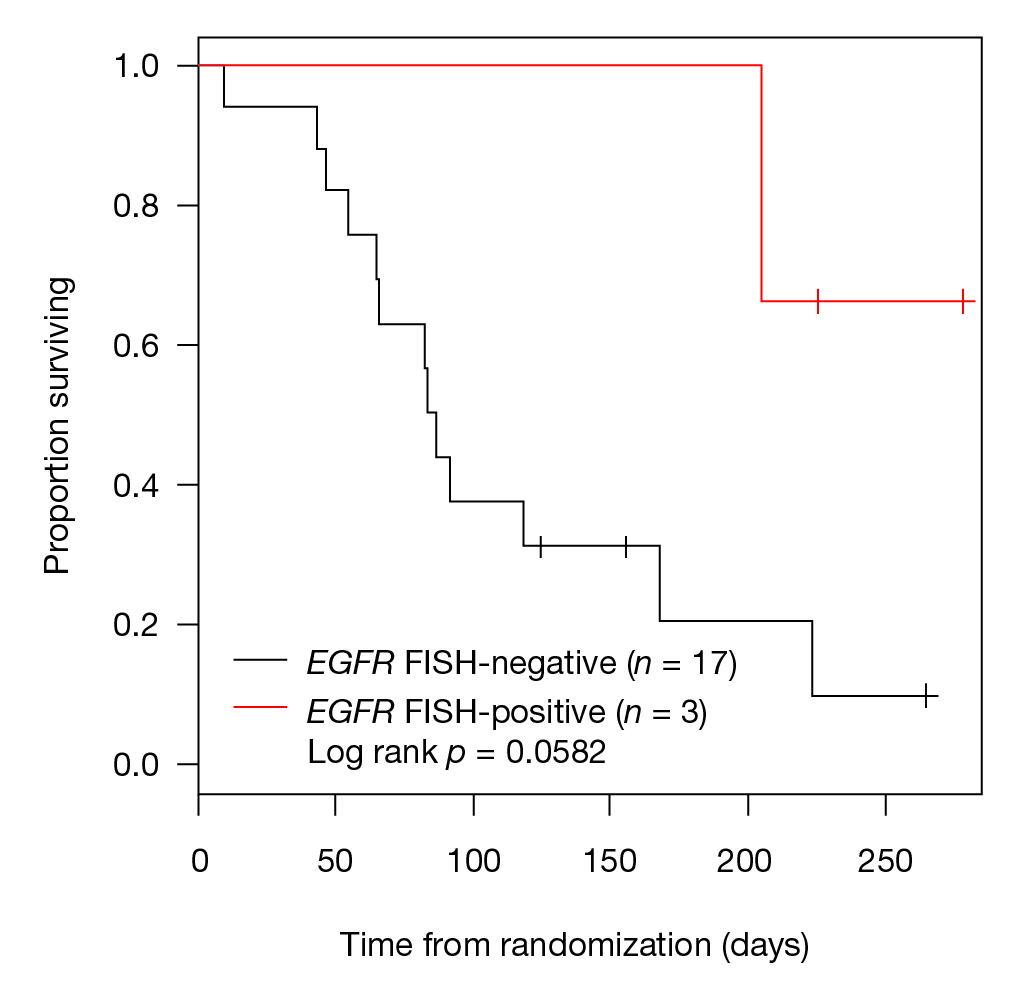

Supplement: Figure S4 — Epidermal growth factor receptor (EGFR) copy number and overall survival (OS) in the lenalidomide monotherapy arm. In the lenalidomide monotherapy arm, OS was shorter for EGFR FISH-negative than for EGFR FISH-positive subjects (median OS: 86 and >277 days, respectively). (TIF) [file pone.0062264.s004.tif]
